# Temporal headache and jaw claudication may be the key for the diagnosis of giant cell arteritis

**DOI:** 10.4317/medoral.22298

**Published:** 2018-04-24

**Authors:** Beatriz Peral-Cagigal, Álvaro Pérez-Villar, Luis-Miguel Redondo-González, Claudia García-Sierra, Marina Morante-Silva, Beatriz Madrigal-Rubiales, Alberto Verrier-Hernández

**Affiliations:** 1 Oral and Maxillofacial Surgery Department. Río Hortega Hospital, Valladolid, Spain; 2 Pathology Department. Río Hortega Hospital, Valladolid, Spain

## Abstract

**Background:**

Temporal artery biopsy (TAB) is a surgical procedure with a low positive yield. The purpose of this study is to determine which variables are the most important in the giant cell arteritis (GCA) diagnosis. The objective of this evaluation is to improve the percentage of positive temporal artery biopsy and if possible, avoid the biopsy in some cases.

**Material and Methods:**

A retrospective clinical study consisted of 90 patients who had undergone TAB at the Río Hortega Hospital (Spain) from January 2009 to December 2016. Clinical findings, erythrocyte sedimentation rates (ESR) and other laboratory parameters, American College of Rheumatology (ACR) criteria for GCA score and biopsy results were recorded.

**Results:**

Nineteen (21.1%) biopsies were positive for GCA. The mean age in positive TAB was 78.6 years old (SD 7.93), and 73.7% were female. Presence of temporal headache (*p* = 0.003), jaw claudication (*p* = 0.001), abnormal artery exploration (*p* = 0.023), elevated erythrocyte sedimentation rate (*p* = 0.035), CRP (*p* = 0.018) and platelets (*p* = 0.042), were significantly associated with GCA. Multivariate logistic regression revealed that the best predictors for the diagnosis of GCA are headache and jaw claudication, adjusted by sex, age, and temporal exploration.

**Conclusions:**

TAB has benefit only for patients who score a 2 or 3 on the ACR criteria for GCA without biopsy. These findings highlight the need for a better diagnostic strategy for patients with suspected temporal arteritis.

** Key words:**Giant cell arteritis, horton arteritis, vasculitis, temporal artery biopsy, jaw claudication, temporal headache.

## Introduction

Giant cell arteritis (GCA), also known as temporal or granulomatous arteritis, described by Horton in 1932, is a relatively common form of blood vessel inflammation, which usually affects people over the age of 50, with peak incidences occurring between the ages of 70 and 80 (1). GCA is a systemic vasculitis that affects large and medium sized arteries, and is most prevalent in the white population of European origin (2). Women are two to three times more susceptible. The inflammatory process leads to vessel scarring, narrowing, severe stenosis, and eventually occlusion (3).

GCA typically causes headaches, tenderness over temporal arteries, jaw claudication (pain with chewing), low-grade fever and systemic upset, but can also be associated with sudden and irreversible sight loss. GCA often progresses rapidly and, if left untreated, leads to severe pain, permanent visual loss, stroke and, in some cases, death. There is an entity called occult GCA which occurs about 20% of the time, and is defined as ocular involvement with absence of systemic symptoms or signs.

Jaw claudication consists in the appearance of pain and tiredness of facial musculature secondary to chewing which goes down with repose. It is due to affectation of the internal maxillary artery and facial artery as well as its branches. This affectation is the most common characteristic of GCA, although it is only present in less than half of the cases based on different studies (4). The importance of this symptom and temporal headache, is that it can be the reason to consult an oral and maxillofacial surgeon. In these cases, we will have to conduct a differential diagnosis with either temporomandibular joint dysfunction or myofascial pain syndrome as well as investigate the presence of other symptoms, signs or analytical alterations which can lead us to suspect that we are encountering a giant cell arteritis case.

Temporal artery biopsy (TAB) is the current gold standard test for establishing the diagnosis, with a high specificity but low sensitivity. It can be misleading in a significant number of cases. Up to 44% of patients with clinical features of GCA have a negative biopsy (5). There are many reasons for this, including the adequacy of the specimen obtained, the duration of glucocorticoid treatment prior to biopsy and the presence of skip lesions (intermittent). However, it is also important to avoid treating those without the condition, because there is a very high incidence of side effects associated with long-term glucocorticoid therapy. Ultrasound and other imaging techniques are emerging as alternative tests to biopsy but have not been widely accepted.

Recent literature suggests that there is now enough evidence that TAB does not change the management of GCA and that the use of imaging modalities, such as ultrasound scan, color doppler sonography and cranial magnetic resonance imaging (MRI) may be sufficient to confirm the diagnosis. Gallium-67 SPECT scintigraphy may be useful in the diagnosis of the disease too, although further studies are needed to confirm this data.

In summary, the management of GCA requires a balance between ensuring that patients with GCA are diagnosed and treated promptly (to avoid complications such as sight loss) and avoiding the burden of unnecessary steroid treatment in people without GCA. Glucocorticoids are the mainstay of treatment and once administered rapid improvement of all clinical manifestations following treatment initiation is characteristic. Thus therapy should not be delayed pending temporal artery biopsy which should be performed as soon as possible. The average duration of treatment is two to three years, but some may develop disease-related or treatment-related complications (osteoporosis, diabetes, hypertension, etc.); careful follow-up and periodic laboratory evaluations with ESR and CRP are required. Prevention, screening, and management of corticosteroid-induced complications are essentials. Failure to respond or development of steroid-related adverse effects are indications to consider immunosuppression with methotrexate.

GCA may result in fatal complications; the major causes of mortality have been vascular: stroke, coronary artery events, rupture of thoracic aortic aneurysms, and aortic dissection.

The purpose of this study is to determine which variables are the most important in the giant cell arteritis (GCA) diagnosis.

## Material and Methods

The design of this study is observational and descriptive, based on diagnostic tests with a benchmark test (gold standard).

A retrospective clinical study consisted of patients who had undergone TAB at the Río Hortega Hospital (Department of Oral and Maxillofacial Surgery, Valladolid, Spain) from January 2009 to December 2016. The same investigator extracted data from all records and collected detailed information using a standardized form. Demographic, laboratory and clinical data were collected. The variables recorded were: age, sex, presence or absence of temporal headache, jaw claudication, visual disturbance (amaurosis fugax, vision loss, diplopia or eye pain), fever, positive biopsy, abnormal temporal artery exploration (decreased pulse or tenderness, pain, thickening and or presence of nodules), previous diagnosis of polymyalgia rheumatica (PMR), and levels of ESR, C-reactive protein (CRP), hemoglobin and platelets. Anemia was defined as hemoglobin level of less than13 g/dl in men and 12 g/dl in women.

**Figure 1:**
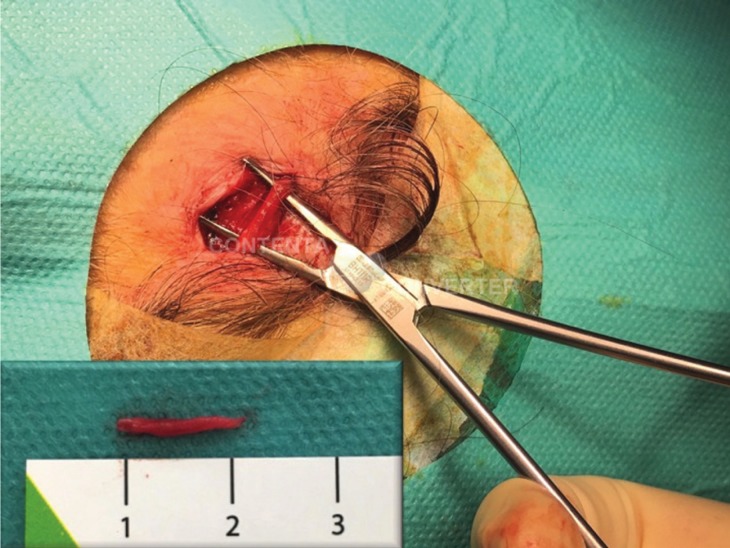
Intraoperative image of a temporal artery biopsy (left side). The most symptomatic artery is usually selected for biopsy and is most likely to show evidence of pathological findings. Size of specimen >1 cm.

**Figure 2:**
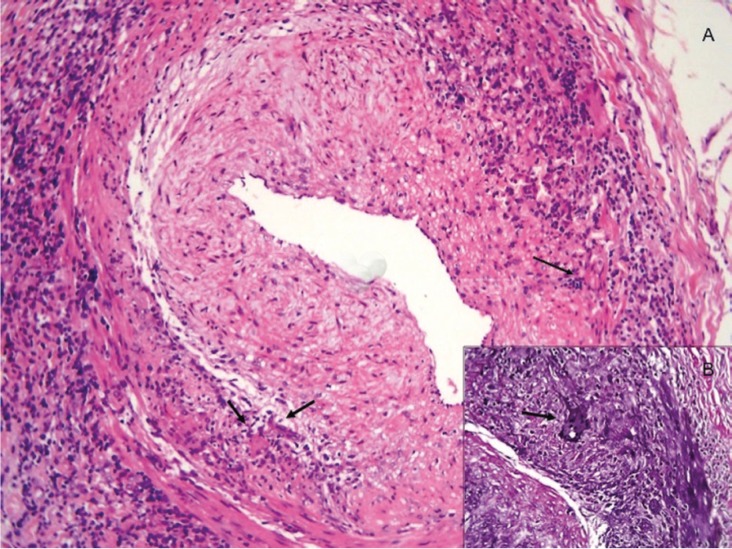
A. Anatomic pathology image (H/E 10 x) of a temporal giant cell arteritis with inflammation of the vessel wall (active arteritis). Adventitious and subintimal fibrosis thickening with decreased light. Inflammatory infiltrates in arterial wall. B. Fragmentation and disruption of the internal elastic lamina with multinucleated giant cells (elastic stain).

All TAB were performed using local anaesthesia and the size of specimens were between 1-2 cm in all patients (Fig. 1). Recent studies suggest that a post-fixation superficial temporal artery biopsy length of 7 to 10 mm is adequate for diagnosing giant cell arteritis (6). Histopathologically (Fig. 2), two patterns were considered diagnostic of giant cell arteritis: those with inflammation of the vessel wall (active arteritis) and those with post-inflammatory alterations (healed arteritis).

All data was entered into Microsoft Excel and statistical analysis was performed using SPSS Version 15.0 (SPSS Inc, 1989-2006). Categorical variables are expressed as a percentage (%) and quantitative variables as mean +/- standard deviation (SD).

Statistical analysis was performed using χ2 analysis or Fisher´s exact test for categorical variables, Student t test for continuous variables, and categorical regression analysis for multivariate analysis. A *p* value less than 0.05 defined statistical significance.

Sensitivitiy, specificity, predictive values, and likelihood ratios were calculated for the association of the various clinical and analytical findings and the presence of a positive biopsy result.

## Results

A total of 90 TAB were performed on 90 patients in our hospital from 2009 to 2016. The mean age was 77.28 years old (SD 9.06), 62.2 % were performed on females and 37.8 % on males. Of the 90 TAB performed, only 19 (21.1%) specimens were reported as positive for GCA, the remaining 71 (78.9 %) specimens reported as negative for GCA. The mean age in positive TAB was 78.6 years old (SD 7.93), and 73.7% were female.

The temporal cephalea was the most frequent symptom in this study, occurring in 68.4% of positive biopsies (statistically significant association, *p*= 0.003), followed by jaw claudication (42.1%, *p* = 0.001) and abnormal exploration (42.1%, *p* = 0.023).

In our study, the mean ESR value in positive TAB cases was 69.16 mm/h (SD 28.9), elevated value was significantly associated with GCA (*p* = 0.035) and mean CRP value was 108.79 mg/l (SD 64.4, *p* =0.018). Therefore, 100% of the positive TAB had abnormal levels of ESR and CRP.

Permanent visual loss has been reported to occur in as high as 15-20% of these patients, making early and correct diagnosis critical (2). In our case, 15.6% of the studied patients had visual alterations and only 26.3% of them had a positive result in the biopsy.

In the present study, positive TAB is also correlated with a lower hemoglobin level (73.7% of patients had anemia) but is not significantly associated with a positive TAB. However there is a statistically significant association with higher platelet count (57.9% of patients, *p* = 0.042).

Before biopsy, 25 of the 90 (27.8%) patients met ACR criteria for GCA with a score of 3 or more. Thirteen (52%) of these had a positive biopsy. Thirteen (14.4%) patients scored ≤ 1 before biopsy, incapable of meeting ACR criteria for GCA; only one (5.3%) of these biopsies was positive. Fifty-two (57.8%) patients scored 2 before biopsy, but only 5 (9.6%) had a positive biopsy ( Table 1). Out of the total positive biopsies, 68.4% met 3 or more criteria before the biopsy was performed. In this study, a score ≥ 3 criteria has a sensitivity of 68.42% and a specificity of 83.1% for GCA.

**Table 1:**
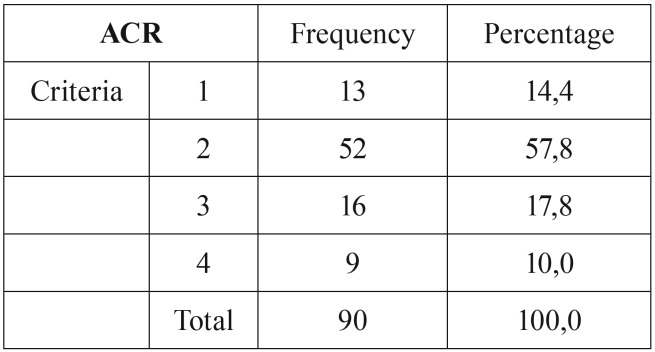
Data table showing the patients distribution according to the number of ACR criteria before performing a temporal artery biopsy.

If we assess the variables gathered in our study, we can observe that having more than 6 variables altered within the 12 studied, the sensitive would be 69,42% and the specificity would be 73,24%.

Multivariate logistic regression revealed that the best predictors for the diagnosis of GCA are headache (OR 6,908. 95% CI 1,716-27,807. *p* = 0.007) and jaw claudication (OR 9,127. 95% CI 1,957-42,578. *p* = 0.005), adjusted by sex (female), age (>78 years) and temporal exploration (abnormal).

## Discussion

The percentage of positive temporal artery biopsy is low, of the 90 TAB performed, only 19 (21.1%) specimens were reported as positive for GCA. However, this appears to be consistent with recently published literature reporting similarly low positive TAB specimen rates of 7-35% (7,8,9). The mean age in positive TAB was 78.6 years old (SD 7.93), and 73.7% were female, similar to other studies (with a peak incidence between the ages of 70 and 80, and more frequent in women).

In our study, the temporal cephalea was the most frequent symptom in positives biopsies (68.4%), followed by jaw claudication and abnormal exploration. In the majority of studies, headache especially located at the temples is the most common symptom (60-90%) and jaw claudication is the most specific symptom (2,9,10).

The two most important labs to order to help make the diagnosis are the ESR and CRP. If they are elevated they may indicate systemic inflammation. Elevation may also be from other causes like infection, malignancy, autoimmune diseases, etc. The combination of ESR and CRP is 97% specific for the diagnosis of GCA according to Hayreh *et al.* CRP was 100% sensitive for detection of GCA and ESR was 92% (11). In our study, 100% of the positive TAB had abnormal levels of ESR and CRP, however they can also be normal. This supports the use of ESR and CRP in the initial diagnosis of GCA.

Polymyalgia rheumatica (PMR) is a clinical syndrome of an unknown etiology, characterized by pain and stiffness, especially in shoulders, neck and hip areas. PMR may be isolated or associated with GCA. In both conditions the erythrocyte sedimentation rate (ESR) and the C-reactive protein (CRP) are elevated, and anemia and thrombocytosis may occur. Five to 15% of PMR patients will be diagnosed with GCA, and 50% of GCA patients will have PMR symptoms (12). In our job, yet only 15.8% of the patients with positive biopsy had a previous PMR diagnosis.

The 1990 American College of Rheumatology (ACR) classification criteria for GCA are based on the following.

-Aged at least 50 years old.

-A new headache.

-Temporal artery abnormality on physical examination (such as tenderness to palpation or decreased pulsation).

-Elevated erythrocyte sedimentation rate (ESR) typically ≥ 50 mm/h.

-Abnormal temporal artery biopsy (TAB) showing vasculitis with mononuclear cell or granulomatous inflammation, usually with giant cells.

The presence of at least three of the five criteria is needed for a positive diagnosis, which yields a sensitivity of 93-94% and a specificity of 91%, although unusual presentations of GCA can be missed. Out of the total positive biopsies, 68.4% met 3 or more criteria before the biopsy was performed. Therefore, like in other studies (1), TAB has only benefited patients who score a 2 or 3 on the ACR criteria for GCA without biopsy.

Since 1932 the conventional gold standard investigation for GCA has been a TAB, showing vasculitis (specificity 100% and sensitivity 15-40%), (7). The characteristic finding of histiocytes, epithelioid and giant cells at the intimal-medial junction is useful in diagnosis, but not always present (e.g. giant cells were found in 75% of positive biopsies in a recent series). Giant cell arteritis affects the vessels in segments, therefore areas of vasculitis may be missed and the histological examination is normal in 10-30% of GCA patients (12). A contralateral biopsy should be considered after a negative initial biopsy if clinical suspicion is high for GCA. However, a negative biopsy can be useful to prevent unnecessary courses of steroids.

TAB is a surgical procedure with a low positive yield. Previous studies have also mentioned the use of algorithms and incorporating the use of ultrasound scan in these algorithms alongside TAB to help improve yield rates of TAB (1). Ultrasound has the highest resolution of all imaging techniques used for the diagnosis of vasculitis. Karassa *et al.* performed a meta-analysis showing that sensitivity of temporal artery duplex ultrasound was 87% and specificity 96% (13).

In conclusion, these findings highlight the need for a better diagnostic strategy for patients with suspected temporal arteritis. Henceforth, more prospective studies which included the studied variables, the ACR criteria and different imaging test must be done in order to improve the percentage of positivity in temporal artery biopsies.
